# Distribution of CMV envelope glycoprotein B, H and N genotypes in infants with congenital cytomegalovirus symptomatic infection

**DOI:** 10.3389/fped.2023.1112645

**Published:** 2023-03-15

**Authors:** Niuniu Dong, Lingfeng Cao, Danni Zheng, Liyun Su, Lijuan Lu, Zuoquan Dong, Menghua Xu, Jin Xu

**Affiliations:** ^1^ Department of Clinical Laboratory, Children's Hospital of Fudan University, National Children's Medical Center, Shanghai, China; ^2^ School of Laboratory Medicine and Life Sciences, Wenzhou Medical University, Wenzhou, China; ^3^ Shanghai Institute of Infectious Disease and Biosecurity, Fudan University, Shanghai, China

**Keywords:** congenital, cytomegalovirus, glycoprotein B, glycoprotein H, glycoprotein N, hearing loss

## Abstract

**Background:**

Cytomegalovirus (CMV) is the leading cause of congenital infections worldwide and contributes to long-term sequelae in neonates and children. CMV envelope glycoproteins play a vital role in virus entry and cell fusion. The association between CMV polymorphisms and clinical outcomes remains controversial. The present study aims to demonstrate the distribution of glycoprotein B (gB), H (gH) and N (gN) genotypes in congenitally CMV (cCMV) infected symptomatic infants and attempts to figure out the association between viral glycoprotein genotypes and clinical outcomes.

**Methods:**

Genotyping of gB, gH and gN was performed in 42 cCMV symptomatic infants and 149 infants with postnatal CMV (pCMV) infection in Children's hospital of Fudan university. Nested PCR, gene sequencing and phylogenetic analyses were used to identify the genotypes.

**Results:**

Our study demonstrated that: 1. The CMV gB1, gH1 and gN1 were the predominant genotypes among symptomatic cCMV infected infants, while gB1, gH1 and gN3a were more prevalent in pCMV group. gH1 genotype has a significant association with symptomatic cCMV infection (*p *= 0.006). 2. No significant correlation was found between CMV genotypes and hearing impairment. However, gH1 was more prevalent among cCMV infected infants with moderate/severe hearing loss although without statistical difference (*p *= 0.130). 3. gB3 was more prevalent among infants with skin petechiae (*p *= 0.049) and found to be associated with an increased risk of skin petechiae (OR = 6.563). The gN4a subtype was significantly associated with chorioretinitis due to cCMV infection (*p *= 0.007). 4. Urine viral loads were not significantly associated with different genotypes or hearing impairment among symptomatic cCMV infected infants.

**Conclusions:**

Our findings demonstrated the overall distribution of gB, gH and gN genotypes in infants with symptomatic cCMV infection in Shanghai for the first time. The findings in our study may suggest a possible association between gH1 genotype and early infancy hearing loss. gB3 genotype was associated with a 6.5-fold increased risk of petechiae while gN4a strongly correlated with chorioretinitis due to cCMV infection. No significant correlation was found between urine viral loads and CMV genotypes or hearing impairment in cCMV infected infants.

## 1. Introduction

Human cytomegalovirus (CMV) belongs to the ß-herpesvirus family infecting most individuals and immunocompromised patients, including the fetus, organ transplant recipients and AIDS population. After primary infection, CMV will establish a lifelong latent infection and could periodically reactivate from latency or reinfection with a new strain (1–4). Cytomegalovirus is the leading cause of congenital infections worldwide while vast majority (90%) of infants with congenital CMV (cCMV) infection is asymptomatic at birth. Approximately 40%–60% of infants with symptomatic and 10%–15% of children with asymptomatic CMV infection will develop long-term neurological sequelae, particularly sensorineural hearing loss (SNHL) (5–12). Congenital CMV infection is the major cause of nongenetic SNHL that could be present at birth or appears later (13). cCMV infection contributes more to the permanent disabilities of infants and young children than other congenital diseases (14–16). Neonates with symptomatic CMV infections are at even higher risk for the adverse neurodevelopmental sequelae and proved to be associated with many severe clinical manifestations (17).

CMV is able to replicate in varieties of cell types, including epithelial cells, endothelial cells, fibroblasts and smooth muscle cells, which facilitate the virus spread within the host and inter-host transmission (18, 19). The broad cell tropism of CMV requires the coordinated interaction of envelope glycoproteins with cell surface receptors (20). The CMV envelope glycoproteins, important targets of virus neutralizing antibodies, are involved in viral entry and cell fusion (21). CMV requires glycoprotein complex gH/gL to fuse with plasma membrane of fibroblasts cells, while entry into epithelial and endothelial cells requires the gH/gL/UL128–131 complex that involves the macropinocytosis and endosomes fusion. Different pathways and glycoprotein complexes play a vital role on the CMV entry into different cell types (22).

Glycoprotein B (gB), encoded by the UL55 gene and classified into 4 genotypes (gB1, gB2, gB3 and gB4), is an abundant and the most highly conserved glycoprotein of CMV. Previous studies have determined that gB is essential for the virus entry and cell-to-cell spread (20, 22–27). The gB variant mediates CMV initial adsorption onto heparin sulfate glycosaminoglycans and interacts with multiple cellular receptors to trigger entry fusion (22, 28). The glycoprotein H (gH), divided into two major genotypes (gH1 and gH2), is an 86-kDa protein and encoded by the UL75 gene. The AD169 and related strains formed gH1 with 743 codons, while Towne and related strains formed gH2 with 742 codons with a deletion of codon 36 (29, 30). gH and gL formed the complex gH/gL, a dimer that was essential to virus entry (31). Besides, the dimer gH/gL together with gO comprise the trimeric glycoprotein complex III (gC-III), or form a pentameric complex which was named as gH/gL/pUL128–131A with three other viral protein UL128, UL130 and UL131. Both of the complexes play a vital role on virus entry as well as inducing virus-neutralizing antibodies (32–38). The highly polymorphic gene UL73 encodes the viral glycoprotein N (gN), a component of the gC-II complex that is involved in virus attachment to the host cell and spread (39–41). The UL73 locus has 7 identified genotypes which were named as gN1, gN2, gN3a, gN3b, gN4a, gN4b and gN4c (42, 43). The UL73 gene possesses highly hypervariable regions (approximately 50% variability), while the nucleotide variations is lower (5%–10%) in gB and gH genes (19, 44). Thus, the polymorphism of gN may enable the virus to evade from neutralizing-antibody response as well as facilitate CMV reinfection in seropositive individuals (45).

Many previous studies have focused on the variability within gB, gH and gN genes owing to their significant role of being major targets of virus neutralizing antibodies. To date, studies on the association between viral glycoprotein polymorphisms and the outcome of symptomatic cCMV infection is controversial (39, 46–51). Hence, the present study aims to determine the distribution of gB, gH and gN genotypes and attempts to ascertain the association between viral glycoprotein polymorphisms and clinical outcomes among symptomatic cCMV infected patients.

## 2. Methods

### 2.1. Study population

A total of 191 infants with symptomatic CMV infection were enrolled from September 2012 to March 2022 at Children's Hospital of Fudan University in Shanghai, China. The geographical origins of the patients were scattered in 16 different districts across the city of Shanghai. Patients were divided into two groups: cCMV infection group and the postnatal CMV (pCMV) infection group. CMV infection was diagnosed by means of real-time PCR performed on urine or plasma. Forty-two infants suffered a cCMV infection (positive test within 21 days from birth), while pCMV infection was diagnosed in 149 infants after three weeks of life (negative test within 21 days of life) (52, 53). The two subpopulations did not differ in their date of infection and geographical area.

The children were classified as having symptomatic infection with any of the following clinical manifestations: jaundice, petechiae, intrauterine growth retardation, hepatosplenomegaly, hepatitis, cholestasis, hearing loss, microcephaly, neurological dysfunction (tremor, hypotonia/hypertonia, or poor sucking reflex), CNS damage in neuroimaging (cerebral calcifications, germinal matrix cysts, ventriculomegaly, and cerebellar hypoplasia), chorioretinitis, pneumonia or laboratory findings, including thrombocytopenia, granulocytopenia, anemia (19, 54).

Our study was reviewed and approved by the Ethics Committee of the Children's Hospital of Fudan University.

### 2.2. Clinical examinations

Demographic data and clinical findings were retrospectively collected from the medical histories of the infants. The clinical manifestations were diagnosed during both hospital stay and outpatient follow-up.

#### 2.2.1. Audiological assessment

All the cCMV infected infants underwent the audiological assessment within the first month of life, by means of Otoacoustic Emission (OAE), Auditory Brainstem Response Audiometry (ABR) and Acoustic impedance test. Hearing thresholds were assessed by means of ABR. Infants with a hearing threshold > 25 decibels (dB) on ABR were considered to have SNHL and scheduled for follow-up of OAE, ABR and acoustic impedance test once a month after discharge. While the cCMV infected infants with a normal hearing on ABR were scheduled for follow-up of OAE, ABR and acoustic impedance test at 3, 6, 12, and 18 months of life after discharge. All the cCMV infected infants were generally followed up until the age of 3 years old. Degree of hearing loss was characterized as normal (≤25 dB), mild (26–40 dB), moderate (41–60 dB), severe (61–80 dB) and profound (over 81 dB) according to the classification of the WHO. Infants diagnosed with monolateral or bilateral hearing loss were classified according to the worst ear threshold.

#### 2.2.2. Ophthalmologic evaluation

Chorioretinitis caused by congenital CMV infection is mainly characterized by yellow-white punctate or patchy exudative changes in the fundus, retinal calcifications, and optic nerve hypoplasia. Ophthalmologic examination was undertaken in the neonatal period and scheduled at 3, 6, 12, and 18 months and up to 3 years of life.

#### 2.2.3. Developmental assessment

Developmental delay in this study refers to infants who did not achieve the appropriate scores in ≥2 performance areas (gross or fine motor, language, cognitive, social and social adjustment, etc.) according to the pediatrician's diagnosis during follow-up. Children's developmental status was investigated by means of the Developmental Screening Test (DST) and the Gesell scales. Griffiths scale is performed in part of clinical patients according to their parents’ opinion. Psychomotor development was assessed at least every 6 months of life and scheduled for a long-term follow-up.

#### 2.2.4. Other definitions

Hyperbilirubinemia was diagnosed when bilirubin value, according to the reference curve of the American Academy of Pediatrics, was over the 95th percentile for the gestational age and for the days of life.

Infantile cholestasis was defined as serum conjugated bilirubin >17.1 *μ*mol/l or direct/total bilirubin ratio >20%.

In the present study, in addition to infants with cyanogenic or non-cyanogenic heart diseases, infants with a Patent Ductus Arteriosus (PDA) and/or a Patent Foramen Ovale (PFO) were considered to have a congenital heart disease (CDH).

### 2.3. CMV detection by real-time PCR

The CMV DNA of urine and plasma were extracted using a commercial diagnostic kit (DaAn Gene Co., Ltd, China) according to the manufacturer's instruction. Saliva samples were extracted using the QIAamp DNA Blood Mini Kit (Qiagen, Germany) according to the manufacturer's protocol.

We used a commercial kit for CMV viral loads measurement (DaAn Gene Co., Ltd, China). The highly conserved non-coding region of IE1 gene in CMV AD169 genome was selected as the amplification target. A 7,500 Real-Time PCR System (Applied Biosystems, Foster City, CA, United States) was used to determine the CMV DNA copy numbers.

### 2.4. CMV glycoprotein genotyping

A nested PCR with two pairs of primers were used to amplify the UL55, UL75 and UL73 gene fragment as described previously (55). All the positive products of nested PCR were sent out for sequencing (Sangon or Tsingke Biotechnology Co., Ltd.). The phylogenetic analysis was conducted by MEGA 11.0 using the neighbor-joining (NJ) method (1,000 bootstrap replications for branch support).

### 2.5. Statistical analysis

All statistical analyses were performed using the SPSS 25.0 software. The data were analyzed using descriptive statistics, including the median, range, and 95% confidence intervals (CIs). Continuous variables are described as medians and categorical data as percentages. The *χ*2 test or Fisher's exact test were used to compare the proportions of categorical variables. Independent group *t*-test was performed on the continuous variables that were normally distributed, while the Mann-Whitney *U* test or Kruskal-Wallis test was used for continuous variables not normally distributed. Two-sided *p*-values of less than 0.05 were considered to be statistically significant.

## 3. Results

### 3.1. Study population

Forty-two newborns (26 males and 16 females) with symptomatic cCMV infection were enrolled in the study. The 149 symptomatic pCMV infection group was consisted of 93 males and 56 females. The median age of cCMV group was 9 days (range 0.13–21 days), while the median age of pCMV infected infants was 3 months (range 0.87–48 months).

In the present study, all the enrolled infants had a symptomatic CMV infection. The distribution and frequency of clinical manifestations in symptomatic neonates with cCMV infection are as follows: Hepatosplenomegaly (4/42, 9.5%), hyperbilirubinemia (25/42, 59.5%), infantile cholestasis (5/42, 11.9%), pneumonia (8/42, 19.0%), thrombocytopenia (16/42, 38.1%), petechiae (10/42, 23.8%), anemia (15/42, 35.7%), neonatal respiratory distress syndrome (NRDS) (7/42, 16.7%), chorioretinitis (12/38, 31.6%), hearing loss (21/39, 53.8%), developmental delay (4/42, 9.5%), congenital heart disease (CHD) (30/42, 71.4%), ventriculomegaly (9/39, 23.1%) and microcephaly (1/42, 2.4%).

### 3.2. Genotype distribution and prevalence of CMV variants

**Figure 1:**
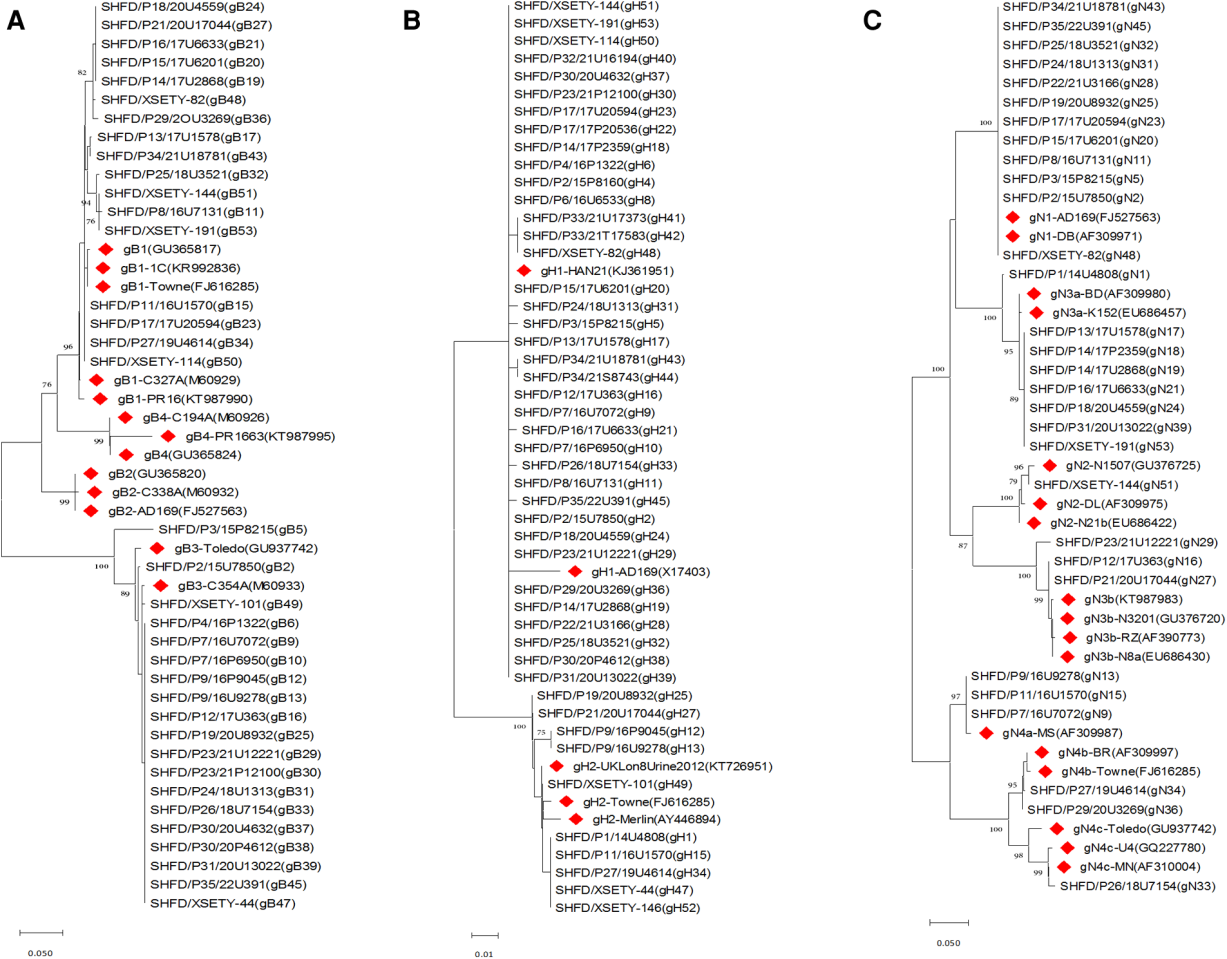
Phylogenetic trees of CMV gB, gH and gN genotypes among symptomatic cCMV infected infants. Phylogenetic trees were generated using MEGA software with method of neighbor-joining and branch supported with 1,000 bootstrap iterations. Reference sequences from GenBank were identified by accession number with red diamonds. (**A**) Phylogenetic trees of gB genotypes. (**B**) Phylogenetic trees of gH genotypes. (**C**) Phylogenetic trees of gN genotypes.

52 specimens from 42 symptomatic cCMV infected neonates were analysed to determine the gB, gH and gN genotypes. The UL55 (gB), UL75 (gH) and UL73 (gN) gene were amplified and sequenced successfully for 32/42 (76.2%), 37/42 (88.1%) and 28/42 (66.7%) of the neonates, respectively. Two out of four genotypes of gB were represented and distributed as follows: gB1 in 17/32 (53.1%) and gB3 in 15/32 (46.9%) of infants. gB2 and gB4 were absent in this group (Figure 1A). With regard to UL75 gene, gH1 was detected in 29/37 (78.4%) of the infants followed by gH2 in 8/37 (21.6%, Figure 1B). UL73 genotyping was accomplished in 28 newborns with gN1 (12/28, 42.9%) being the most dominant genotype, followed by gN3a (6/28, 21.4%), gN3b (3/28, 10.7%), gN4a (3/28, 10.7%), gN4b (2/28, 7.1%), gN4c (1/28, 3.6%) and gN2 (1/28, 3.6%). All seven genotypes of gN were represented in this cohort although some types were available in very small numbers (Figure 1C). All the expected genotypes were present except gB2 and gB4 among symptomatic cCMV infection group (Table 1).

**Table 1:** Distribution of gB, gH and gN genotypes among symptomatic cCMV and pCMV infected children.

Genotypes	cCMV infection (*n*, %)	pCMV infection (*n*, %)	*p*-value
gB1	17 (53.1)	62 (54.4)	1.000
gB3	15 (46.9)	51 (44.7)	
gB4	0	1 (0.9)	
Total	32	114	
gH1	29 (78.4)	78 (53.4)	0.006*
gH2	8 (21.6)	68 (46.6)	
Total	37	146	
gN1	12 (42.9)	16 (17.4)	0.126
gN2	1 (3.6)	2 (2.2)	
gN3a	6 (21.4)	26 (28.3)	
gN3b	3 (10.7)	7 (7.6)	
gN4a	3 (10.7)	14 (15.2)	
gN4b	2 (7.1)	16 (17.4)	
gN4c	1 (3.6)	11 (12.0)	
Total	28	92	

cCMV infection: congenital CMV infection; pCMV infection: postnatal CMV infection.

* *p* < 0.05 was considered of significant difference between the two groups.

Out of 149 infants with symptomatic pCMV infection, 76.5% (114/149) of gB, 98.0% (146/149) of gH, and 61.7% (92/149) of gN accomplished the genotyping. The CMV gB1 (62/114, 54.4%), gB3 (51/114, 44.7%) and gB4 (1/114, 0.9%) were detected except the genotype of gB2. gH1 was found in 78/146 (53.4%) of the infected infants followed by gH2 (68/146, 46.6%). All seven genotypes of gN were presented in this group and distributed as follows: gN1 in 16/92 (17.4%), gN2 in 2/92 (2.2%), gN3a in 26/92 (28.3%), gN3b in 7/92 (7.6%), gN4a in 14/92 (15.2%), gN4b in 16/92 (17.4%) and gN4c in 11/92 (12.0%) of infants, respectively (Table 1).

Genotype distribution of gH was significantly different between the cCMV and pCMV groups. The predominance of gH1 in cCMV infected infants was much more pronounced compared to the pCMV infected children (*p *= 0.006). There was no statistical difference in the distribution of gB and gN genotypes between the two groups (Table 1).

### 3.3. CMV glycoprotein polymorphisms and neonatal hearing loss

The hearing test was available in 39/42 (93%) of symptomatic cCMV infected neonates in our study. 18 patients (46%) passed the hearing test while 21 cases (54%) failed. Of the 21 infants who failed the hearing test, the gB genotyping was completed for 15 infants with 7 (46.7%) for gB1 and 8 (53.3%) for gB3. The gH genotyping could be accomplished in 19 newborns with 15 (78.9%) for gH1 and 4 (21.1%) for gH2. Genotyping of gN was completed in 13 infants and distributed as follows: gN1 in 5/13 (38.5%), gN2 in 1/13 (7.7%), gN3a in 3/13 (23.1%), gN3b in 1/13 (7.7%), gN4a in 2/13 (15.4%) and gN4c in 1/13 (7.7%). There was no statistical difference in the distribution of gB, gH and gN genotypes among symptomatic cCMV infected neonates with or without hearing loss (Table 2).

**Table 2:** Distributions of gB, gH and gN genotypes among symptomatic cCMV infected neonates with or without hearing loss.

Genotypes	Pass Hearing Test (*n*, %)	Fail Hearing Test (*n*, %)	*p*-value
gB1	8 (57.1)	7 (46.7)	0.715
gB3	6 (42.9)	8 (53.3)	
Total	14	15	
gH1	11 (73.3)	15 (78.9)	1.000
gH2	4 (26.7)	4 (21.1)	
Total	15	19	
gN1	5 (41.7)	5 (38.5)	0.841
gN2	0	1 (7.7)	
gN3a	3 (25.0)	3 (23.1)	
gN3b	1 (8.3)	1 (7.7)	
gN4a	1 (8.3)	2 (15.4)	
gN4b	2 (16.7)	0	
gN4c	0	1 (7.7)	
Total	12	13	

To further explore the hearing impairment among neonates, we analyzed the gB, gH and gN genotypes depending on the hearing loss severity among infants who failed the test (Table 3). We found that among these infants with hearing impairment, 11 out of 13 ears from 8 newborns with moderate/severe hearing loss all exhibited the gH1 genotype rather than gH2. Two ears from one child with moderate hearing loss failed the sequencing. gH1 was the predominant genotype among symptomatic cCMV infected infants with moderate/severe hearing impairment although without statistical difference (*p *= 0.130, Table 3).

**Table 3:** Distribution of gB, gH and gN genotypes among symptomatic cCMV infected children with different hearing loss severities.

Genotypes	Normal (*n*, %)	Hearing loss severity, *n* (%)		*p*-value
		Mild (26-40 dB)	Moderate/severe (>41 dB)	
gB1	19 (57.6)	4 (44.4)	3 (37.5)	0.588
gB3	14 (42.4)	5 (55.6)	5 (62.5)	
Total	33	9	8	
gH1	28 (75.7)	7 (70.0)	11 (100)	0.130
gH2	9 (24.3)	3 (30.0)	0	
Total	37	10	11	
gN1	11 (37.9)	2 (28.6)	3 (37.5)	0.285
gN2	1 (3.4)	0	0	
gN3a	8 (27.6)	2 (28.6)	1 (12.5)	
gN3b	2 (6.9)	2 (28.6)	0	
gN4a	3 (10.3)	1 (14.3)	2 (25.0)	
gN4b	4 (13.8)	0	0	
gN4c	0	0	2 (25.0)	
Total	29	7	8	

### 3.4. Viral loads

The CMV DNA was detected in all the 42 newborns with confirmed cCMV infection. The log values of CMV DNA concentration in 38 urine specimens ranged from 3.03 to 7.64 copies/ml (median 5.34 copies/ml; mean 5.34 ± 1.31 copies/ml). In 13 blood samples, the viral loads ranged from 3.13 to 5.94 copies/ml (median 4.41 copies/mL; mean 4.47 ± 0.87 copies/ml). The median viral loads in urine samples were significantly higher than those in blood samples (*p *= 0.03, data not shown). We did not find the association between CMV gB, gH and gN genotypes with urine viral loads among symptomatic infants (Table 4).

**Table 4:** Comparison of viral loads of gB, gH and gN genotypes in urine samples in infants with symptomatic cCMV infection.

Genotypes	Urine Viral load (Log) copies/ml	*p*-value
gB genotypes		0.789
gB1	17 (5.50, 4.18–7.40)	
gB3	13 (6.12, 3.33–7.43)	
gH genotypes		0.305
gH1	27 (5.34, 3.03–7.43)	
gH2	8 (6.12, 3.33–7.64)	
gN genotypes		0.735
gN1	11 (5.50, 3.33–7.40)	
gN2	1 (6.72)	
gN3a	6 (5.03, 4.43–6.84)	
gN3b	3 (5.16, 5.12–7.43)	
gN4a	3 (6.45, 5.34–7.15)	
gN4b	2 (5.05, 4.18–5.92)	
gN4c	1 (5.32)	

Besides, we compared the urine viral loads in cCMV infected infants with or without hearing impairment. The log values of CMV DNA concentration in 16 infants with normal hearing ranged from 3.33 to 7.43 copies/ml (median 5.24 copies/ml; mean 5.12 ± 1.16 copies/ml). In 19 infants with hearing impairment, the viral loads ranged from 3.03 to 7.64 copies/ml (median 5.58 copies/ml; mean 5.42 ± 1.46 copies/ml). The hearing impairment group has a higher urine viral loads than the normal hearing group although without statistical difference (*p *= 0.512, data not shown).

### 3.5. gB, gH, gN genotypes vs. clinical indicators and cCMV-related symptoms/outcomes

In the present study, no significant association was found between a specific gB, gH or gN genotype and different clinical indicators (Table 5). gB3 (7/15, 46.7%) was more prevalent among newborns with skin petechiae compared with gB1 (2/17, 11.8%; *p *= 0.049, Table 6). Infection with gB3 genotype was associated with a 6.5-fold increased risk of skin petechiae (OR= 6.563 [95% CI, 1.095–39.324]; *p *= 0.049, data not shown). Besides, the gN4a subtype was significantly correlated with chorioretinitis diagnosis (3/3, 100%; *p *= 0.007, Table 6).

**Table 5:** Distribution of gB, gH and gN genotypes in different clinical indicators among infants with symptomatic cCMV infection.

Clinical Indicators	gB1 (*n* = 17)	gB3 (*n* = 15)	gH1 (*n* = 29)	gH2 (*n* = 8)	gN1 (*n* = 12)	gN2 (*n* = 1)	gN3a (*n* = 6)	gN3b (*n* = 3)	gN4a (*n* = 3)	gN4b (n = 2)	gN4c (*n* = 1)
Male	9 (52.9)	10 (66.7)	19 (65.5)	3 (37.5)	6 (50)	1 (100)	5 (83.3)	1 (33.3)	1 (33.3)	1 (50)	1 (100)
Age	9 (0.88–20)	8 (0.13–17)	9 (0.88–21)	12 (0.1–16)	9 (0.88–18)	1.63	9.5 (1–15)	4 (2–14)	9 (5–12)	16.5 (13–20)	4
BW	2,150 (1180–3150)	2,650 (1470–3800)	2,160 (1180–3800)	2,555 (1470–3050)	2,035 (1470–3400)	2150	2,110 (1180–3800)	2,250 (1800–2960)	2,650 (2500–3050)	2917.5 (2720–3115)	2740
GA	36.1 (32–41)	38 (28.7–42.6)	36.1 (28.7–41)	37.7 (29.1–42.6)	36 (29.1–41)	36.7	34.4 (32–38.3)	34.9 (28.7–39.3)	38 (37.3–39.3)	37.7 (37.4–37.9)	37.1
CM	15 (88.2)	12 (85.7)	21 (75.0)	6 (85.7)	8 (72.7)	1 (100)	5 (83.3)	3 (100)	3 (100)	2 (100)	0
CG	202.3 (25.6–500)	301.2 (15.64–500)	259.4 (15.6–500)	284.5 (55.4–500)	318.2 (48.8–500)	25.6	150.2 (40.9–367.7)	170.8 (55.4–438.4)	284.5 (223.2–500)	333.8 (192.6–475)	15.6
AST	36.7 (12–366)	38 (16–701.4)	37 (12–701.4)	33.9 (19–162)	36.5 (13–366)	230	28.3 (12–87)	70.3 (33–701.4)	39 (24–40)	41.4 (36.7–46)	168
ALT	11 (2–64)	12.4 (6–242)	11 (2–242)	11.2 (8–33)	11.7 (3–242)	52	7.5 (2–11)	12.4 (6–224.1)	14 (8–19)	21.9 (16–27.7)	39
TBil	123.2 (17.7–261.8)	85.7 (16.5–294.9)	109.5 (16.5–294.9)	124 (16.6–219)	112.1 (30.1–222.2)	261.8	102.7 (32.5–294.9)	137.3 (51.5–156.7)	27.2 (16.5–219)	81.5 (17.7–145.3)	109.5
DBil	12.4 (4.6–173.3)	15.9 (7.3–81.7)	15.2 (5.9–173.3)	9.6 (4.6–112.4)	16.3 (5.9–81.7)	173.3	11.1 (7.7–15.2)	15.9 (4.6–17.2)	7.6 (7.6–14.4)	10.1 (8.5–11.6)	11.6
TBA	13 (5.5–82.5)	10.5 (2.5–63.5)	15.2 (5.5–82.5)	7.3 (2.5–104.6)	15.1 (7.1–77.5)	19.2	10.5 (5.5–82.5)	6.5 (5.6–30.6)	7 (2.5–44.1)	32.5 (8.8–56.2)	12.9
APTT	45.8 (32.5–90.6)	49.8 (33.1–63.8)	49.9 (32.5–90.6)	48 (34.6-61.3)	52.3 (45.3–90.6)	50	40 (40–73.8)	50.2 (49.8–50.6)	33.9 (33.1–34.6)	32.5	/
INR	1.3 (0.9–1.98)	1.53 (0.92–1.75)	1.3 (0.9–1.98)	1.0 (0.92–1.15)	1.25 (1.08–1.98)	1.57	1.42 (0.9–1.96)	1.64 (1.53–1.75)	0.96 (0.92–0.99)	1.11	/
PLT	218 (19–499)	127 (23–399)	182 (19–432)	235.5 (105–499)	167 (21–432)	19	270.5 (164–301)	166 (60–361)	127 (53–203)	393 (287–499)	399

BW, birth wight; GA, gestational age; CM, CMV-IgM pos; CG, CMV-IgG; AST, aspartate aminotransferase; ALT, alanine aminotransferase; TBil, total bilirubin; DBil, direct bilirubin; TBA, total bile acid; APTT, activated partial thromboplastin time; INR, international normalized Ratio; PLT, platelet.

The clinical indicator of age (days), BW (g), GA (weeks), CG, AST, ALT, TBil, DBil, TBA, APTT, INR and PLT were shown as median (min-max). Gender (male) and CM (positive) were shown as (*n*, %).

**Table 6:** Association between the gB, gH and gN genotypes and clinical symptoms/outcomes among infants with symptomatic cCMV infection.

Findings	Number of patients with CMV gB, gH, gN genotypes, *n* (%)													
	gB1	gB3	*p-*value	gH1	gH2	*p-*value	gN1	gN2	gN3a	gN3b	gN4a	gN4b	gN4c	*p-*value
Anemia	4 (23.5)	6 (40)	0.450	9 (31.0)	4 (50.0)	0.413	4 (33.3)	0	2 (33.3)	2 (66.7)	2 (66.7)	0	0	0.732
Ventriculomegaly	2 (12.5)	5 (33.5)	0.220	7 (25.0)	2 (28.6)	1.000	4 (36.4)	0	0	2 (66.7)	2 (66.7)	0	0	0.206
Microcephaly	1 (5.9)	0	1.000	0	1 (12.5)	0.216	0	0	0	1 (33.3)	0	0	0	0.357
Hearing loss	7 (46.7)	8 (57.1)	0.573	15 (57.7)	4 (50.0)	1.000	5 (50.0)	1 (100)	3 (50.0)	1 (50.0)	2 (66.7)	0	1 (100)	0.841
Chorioretinitis	5 (35.7)	6 (40)	0.812	8 (30.8)	3 (37.5)	1.000	1 (10.0)	0	4 (66.7)	0	3 (100)	1 (50)	0	0.007*
Developmental delay	2 (12.5)	2 (13.3)	1.000	2 (7.1)	2 (25.0)	0.207	0	0	1 (20.0)	1 (33.3)	2 (66.7)	0	0	0.081
Thrombocytopenia	6 (35.3)	8 (53.3)	0.305	14 (48.3)	2 (25.0)	0.423	6 (50)	1 (100)	0	2 (66.7)	2 (66.7)	1 (50)	0	0.120
Hepatosplenomegaly	2 (11.8)	1 (6.7)	1.000	3 (10.3)	1 (12.5)	1.000	1 (8.3)	0	1 (16.7)	0	0	0	0	1.000
Petechiae	2 (11.8)	7 (46.7)	0.049*	7 (24.1)	3 (37.5)	0.655	2 (16.7)	0	1 (16.7)	2 (66.7)	2 (66.7)	0	0	0.286
NRDS	1 (5.9)	2 (13.3)	0.589	3 (10.3)	1 (12.5)	1.000	2 (16.7)	0	1 (16.7)	1 (33.3)	0	0	0	0.936
Infantile cholestasis	2 (11.8)	2 (13.3)	1.000	4 (13.8)	1 (12.5)	1.000	2 (16.7)	0	0	0	0	0	0	0.810
Pneumonia	3 (17.6)	5 (33.3)	0.423	8 (27.6)	0	0.160	2 (16.7)	0	1 (16.7)	1 (33.3)	1 (33.3)	0	1 (100)	0.569
CHD	13 (76.5)	10 (66.7)	0.699	21 (72.4)	5 (62.5)	0.672	10 (83.3)	0	5 (83.3)	2 (66.7)	1 (33.3)	2 (100)	1 (100)	0.286
Hyperbilirubinemia	9 (52.9)	7 (46.7)	0.723	16 (55.2)	4 (50.0)	1.000	6 (50.0)	0	4 (66.7)	3 (100)	1 (33.3)	0	1 (100)	0.268
Total	17	15	/	29	8	/	12	1	6	3	3	2	1	/

* *p* < 0.05 was considered of significant difference between the groups. NRDS, neonatal respiratory distress syndrome; CHD, congenital heart disease.

## 4. Discussion

Congenital cytomegalovirus infection in newborns may cause severe sequelae such as central nervous system (CNS) damage, SNHL, intellectual disability and various congenital malformations. Infants born with symptomatic cCMV infection are at a higher risk for developing adverse long-term outcomes. The present study focused on the polymorphisms of UL55, UL75 and UL73 gene in infants with symptomatic CMV infection and evaluated the possible association between CMV genotypes and clinical outcomes in neonates with symptomatic cCMV infection.

Overall, our study indicated that gB1 (17/32, 53.1%), gH1 (29/37, 78.4%) and gN1 (12/28, 42.9%) were the most common genotypes among cCMV group, while gB1 (62/114, 54.4%), gH1 (78/146, 53.4%) and gN3a (26/92, 28.3%) were more prevalent in pCMV group. The cCMV genotypic distribution in our study was similar to the reports from previous studies. A research from Puhakka et al. (56) in cCMV infected infants confirmed our findings: they demonstrated that gB1 was the most common genotype (19/37, 51%) followed by gB3 (24%), gB2 (19%) and gB4 (5%). The most common genotype of gN was gN1 (7/24, 29%) followed by gN4c (25%), gN3b (21%), gN4a (13%), gN3a and gN4b (8%). Another cohort from Pakistan came to a similar result demonstrating gB1 (4/10, 40%), gH1 (7/11, 63.7%) and gN1 (3/15, 20%) to be the most common genotypes among cCMV infected infants (57). Previous studies from different geographical regions further indicated that the gB1 (47, 58–64), gH1 (19, 65) and gN1 (64) were the most prevalent genotypes in newborns with cCMV infection.

To date, the dominant genotypes of UL55, UL75 and UL73 gene among different cCMV cohorts worldwide are still controversial. Paradowska et al. demonstrated that the gB2 genotype was prevalent in Polish newborns with symptomatic cCMV infection (48, 66). However, Sarkar et al. (17) demonstrated that gB1 was the most widespread genotype among Indian neonates with symptomatic cCMV infection. Another two cohorts also confirmed our findings that gB1 was the most common genotype in neonates and infants with symptomatic cCMV infection (57, 67). Moreover, a cohort from India (68) revealed that gB3 was the most prevalent genotype in symptomatic infants. The controversial results were also observed in gH and gN genotypes. Pati et al. showed that the most predominant genotype among cCMV infected infants in American individuals were gH2 (59%) and gN3a (27%), respectively (62). Contrary to our results, Paradowska et al. (69) demonstrated that the gH2 variant occurred more frequently compared with gH1 in newborns with symptomatic cCMV infection. It was observed in a study of Pignatelli et al. that gN4a (66.7%), gN4b (60%) and gN4c (52.9%) were more prevalent among symptomatic newborns in Italy (70). Furthermore, Paradowska et al. (71) demonstrated that gN3b (14/42, 33.3%), gN4b (12/42, 28.6%), and gN4c (11/42, 26.2%) were more prevalent and supported a potential role of gN as the virological marker in newborns with symptomatic CMV infection. All these discrepancies may be attributed to geographical distribution, population/sample selection bias, genotyping method and/or CMV tissue tropism.

The majority of previous studies indicated that the genotypic distribution was similar between cCMV and pCMV infected infants (19, 66, 69). A study in Italy (44) found a significant association of gN4c and gO3 genotype with congenital infection (*p *= 0.037 and 0.045, respectively). In our study, no significant difference was observed on the distribution of gB and gN genotype between the two groups. Notably, we revealed that the gH1 genotype has a significant association with symptomatic cCMV infection for the first time (*p *= 0.006).

In this cohort, we were not able to demonstrate a significant association between CMV glycoprotein polymorphisms and neonatal hearing loss. However, infants who suffered moderate or more severe hearing loss all exhibited the gH1 genotype. Paradowska et al. demonstrated for the first time that SNHL has a significant correlation with gH1 genotype (*p *= 0.032) and suggest gH2 could diminish the risk of hearing impairment in infants (69). These results were confirmed by the same group in a larger cohort (19). A chinese study (65) also found a predominance of gH1 genotype in cCMV children suffering SNHL. We observed in a previous study that the gH1 genotype was predominant in infants with active CMV infection, while gH2 was more prevalent in children with latent infection (55). All these observations suggest that gH1 may be involved in early infancy hearing loss due to cCMV infection. However, a larger cohort of neonates is needed to clarify the trends observed in our study.

We observed that the median viral load in urine was significantly higher than that in blood samples, as other author also reported previously (19). We did not find a significant association between CMV genotypes and urine viral loads. The hearing impairment group had a higher urine viral loads when compared to unaffected group but without statistical significance (*p *= 0.512). Previous studies from different cohorts have demonstrated that a higher CMV DNAemia during early infancy was associated with long-term sequelae (72–75). Ross et al. also indicated that a virus burden of <3500 ge/ml in blood is found to be at lower risk of hearing loss in asymptomatic cCMV infected infants (76). We did not analyze the correlation between blood viral loads and CMV genotypes or hearing impairment due to the limited sample size. A larger cohort is needed to illustrate the correlation between blood viral loads and CMV genotypes or hearing impairment.

Previous reports have demonstrated with controversial results that the gN4 genotype represented the most virulent variant and was associated with severe manifestations compared with gN1 and gN3a (70, 77,78). These studies indicated that gN1 or gN3a could reduce the risk of CMV related sequelae 5 folds, whereas the gN4 genotypes increase the risk of sequelae 8 folds. Paradowska et al. (71) confirmed these findings and demonstrated that gN4 genotype was significantly associated with neurological disorders (*p *= 0.045). They suggest that gN2 or gN4 genotypes might be an indicator of serious manifestation in children, while gN1 and gN3b might represent less virulent strains. Similar to their findings, we found that the gN4a subtype was associated significantly with chorioretinitis (*p *= 0.007) due to cCMV infection.

In the present study, CHD incidence (approximately 70%) is much higher than currently reported. Two reasons may explain the huge incidence of CHD in this study. First, the majority of our population consisted of preterm infant and thus the rate of PDA at the first echocardiographic screening was high. Second, PFO was also included between CHD and it is well known that PFO has an extremely high incidence in early infancy. Due to the lack of the results of the echocardiographic follow up proving, in all likelyhood, the resolution of a great part of these cases, all PDA and PFO cases were then included as CHD.

To date, this is the first study focusing on three CMV genotypes and trying to figure out the association between the genotypes and symptomatic cCMV symptoms/outcomes in Shanghai. However, there are several limitations in our study as well. Firstly, the cCMV sample size is small and the results observed in our study should be interpreted with caution. Additional investigation with a larger population of symptomatic cCMV infection is needed to confirm our findings. Secondly, the successful sequencing rate was not satisfactory. Some of the specimens have been stored for a long time and the viral loads may degrade to some extent. The primer specificity of gB, gH and gN may vary and cause the different sequencing results (gH > gB > gN). Further, a successful sequencing greatly depends on the type of sample used. In our study, urine and saliva samples achieved a higher rate of sequencing than blood samples. Thirdly, we did not have the data on mixed infections due to the limitation of the method. We used the direct PCR-sequencing method and the primers were designed to amplify the variable regions of CMV genome. This genotyping method allows detection of the predominant genotype in single specimen and the mixed infections may have been missed. As genotypic tests can only detect viral genotypes when these comprise at least 20%–25% of the total viral population (66), it is likely that only the dominant genotypes were detected. Finally, it should be underlined that we are focusing on the symptomatic cCMV infection population. The absence of a population of asymptomatic infants limits the stratification of disease risk (based on different genotypes) only to the type of disease rather than to the possible occurrence of the disease itself.

In summary, our study showed that CMV gB1, gH1 and gN1 were the predominant genotypes among symptomatic cCMV infected infants in Shanghai. The findings in our study suggest a possible correlation between gH1 genotype and SNHL due to cCMV infection. The gB3 genotype was associated with a 6.5-fold increased risk of skin petechiae and gN4a significantly correlated with chorioretinitis. No significant association was established between CMV genotypes or hearing impairment and the urine viral loads.

## Data Availability

The data presented in the study are deposited in the GenBank repository, accession number OQ453042-OQ453154.
